# Correlation of serum KL-6 and CC16 levels with neurodevelopmental outcome in premature infants at 12 months corrected age

**DOI:** 10.1038/srep08121

**Published:** 2015-01-29

**Authors:** Zhiqun Zhang, Hui Lu, Yunxia Zhu, Junhua Xiang, Xianmei Huang

**Affiliations:** 1 Division of Neonatology, Department of Pediatrics, Hangzhou First People's Hospital, Zhejiang, China; 2 Department of Pediatrics, Hangzhou First People's Hospital, Zhejiang, China

## Abstract

The aim of this study was to evaluate KL-6 and CC16 levels and their correlation with neurodevelopmental outcome among very low birth weight pre-term infants at 12 months corrected age. This prospective cohort study was performed from 2011 to 2013 by enrolling pre-term neonates of gestational age ≤ 32 weeks and birth weight ≤ 1500 g. Serum KL-6 and CC16 levels were determined 7 days after birth and their correlation with neurodevelopment was evaluated using Gesell Mental Developmental Scales. Of the 86 eligible pre-term infants, 63 completed follow-up, of which 15 had bronchopulmonary dysplasia. At 12 months corrected age, 49 infants had favorable outcomes and 14 infants had poor neurodevelopmental outcome. KL-6 levels were higher and CC16 levels were lower in infants with poor neurodevelopmental outcome compared with those infants who had favourable neurodevelopmental outcome. Serum KL-6 levels less than 90.0 ng/ml and CC16 levels greater than 320.0 pg/ml at 7 days of life were found to be predictive of a favourable outcome at 12 months corrected age. These biological markers could predict neurodevelopmental outcome at 12 months corrected age in very low birth weight premature infants, and help the clinician plan early therapeutic interventions to minimize or avoid poor neurodevelopmental outcome.

KL-6 is preferentially expressed on alveolar type II cells in human lungs, and is a marker of specific lung injury^1^,^2^. Following alveolar injury, regenerating type II cells strongly express KL-6 antigen and this can lead to increased plasma KL-6 levels. Markedly increased KL-6 levels in plasma have been reported in patients with various interstitial lung diseases^3^,^4^. The predictive characteristics of increased levels of KL-6 in serum and tracheal aspirate at an early age for the development of bronchopulmonary dysplasia (BPD) in pre-term infants, have been recognized^5^,^6^,^7^,^8^. CC16, a lung-specific protein produced by the tracheobronchial epithelium where non-ciliated Clara cells are predominant, is believed to increase in the circulating blood of subjects with pathological conditions that are characterized by increased permeability of the alveolar–capillary barrier^9^. CC16 has been shown to have anti-inflammatory properties^10^ and has been found at lower concentrations in pre-term infants. Such levels have independently predicted the development of BPD^11^,^12^,^13^,^14^.

Recent studies have shown that infants with BPD were re-hospitalized more frequently due to respiratory disease, compared with infants without BPD^15^,^16^. Furthermore, BPD was found to have a direct adverse effect on the immature brain, particularly cerebral white matter, and on neurodevelopmental outcome^17^,^18^,^19^,^20^. Experimental and clinical studies suggest that inflammatory processes play a role in the etiology and pathogenesis of periventricular leukomalacia^21^,^22^ and white matter damage, leading to adverse neurodevelopmental outcome^23^,^24^. KL-6 and CC16 were identified as early markers for BPD and mirror the intensity of perinatal inflammatory activity. It is not well defined whether these proteins have a role as early markers for predicting long-term outcome such as neurodevelopmental outcome.

To that end, we studied the relationship of early KL-6 and CC16 levels in serum with neurodevelopmental outcome at 12 months corrected age in a prospective cohort of 63 pre-term infants, with a gestational age (GA) of less than 32 weeks. We determined the neurodevelopmental outcome at 12 months of corrected age using Gesell Mental Developmental Scales.

## Methods

### Study population

This study was approved by the ethics committees of the First People's Hospital in Hangzhou. Informed consent was obtained from the parents of the neonates in the study according to institutional guidelines. Between June 2011 and July 2013, all pre-term neonates with a birth weight (BW) of 600–1,500 g and GA of 25–32 weeks admitted to the neonatal intensive care unit were prospectively enrolled into this study. Neonates with congenital heart diseases, multiple malformations, or documented chromosomal abnormalities were excluded. Also excluded from the final analysis were infants who died of non-respiratory causes, or for whom blood samples were not obtained 7 days post-partum.

### Demographic and Perinatal Data

The following demographic and perinatal characteristics were recorded: gender, birth weight, gestational age, surfactant administration, duration of mechanical ventilation, pregnancy-induced hypertension, chorioamnionitis, pre-natal steroid administration, mode of delivery, Apgar score, and cord blood gas details. We also recorded data on the following complications: bronchopulmonary dysplasia (BPD), necrotizing enterocolitis (NEC) Bell's stage > or = II^25^, retinopathy of prematurity (ROP), bloodstream infections, severe intraventricular haemorrhages (IVH) and periventricular leukomalacia (PVL). BPD was defined in accordance with guidelines of the National Institute of Child Health and Human Development/National Heart, Lung, and Blood Institute Workshop (National Institutes of Health consensus definition) for infants born at gestational age < 32 weeks, i.e. treatment with >21% oxygen for at least 28 d^26^. Severe IVH was defined as blood/echodensity in the cerebral parenchyma, or ventricular enlargement occurring in association with blood/echodensity in the ventricular system (Grade III or IV intracranial hemorrhage)^27^. Decisions on corticosteroid treatment for BPD were made by the attending neonatologist, who was blinded to the study results, and referenced to the policy statement of the American Academy of Pediatrics. Infants on assisted ventilation or oxygen therapy over 2 weeks received low-dose hydrocortisone therapy (1 mg/kg per day) for 5–7 days^28^.

### Blood sampling and plasma KL-6 and CC16 measurements

We obtained heparinized blood samples from infants by venipuncture at 7 days post-partum, for estimation of KL-6 and CC16 levels. The blood samples were immediately centrifuged at 3000 × *g* for 10 min at 4°C to obtain plasma, and then stored at −80°C. The KL-6 and CC16 levels in plasma were measured using a quantitative colorimetric sandwich enzyme-linked immunosorbent assay kit (BlueGene, China) in accordance with the manufacturer's instructions. Each sample was run in duplicate and the mean concentration was calculated. As per the kit, the sensitivities for KL-6 and CC16 were 0.1 ng/mL and 1.0 pg/mL, respectively.

### Assessment of neurodevelopmental outcome

The development schedules used in this study were a set of four timetables devised by Arnold Gesell (1880–1961) at Yale University to evaluate the physical, emotional, and behavioral development of infants, toddlers and pre-schoolers. They describe typical behavior at specific ages in the following areas: ability to adapt, motor functioning, use of language, and social interaction^29^. Here, the intellectual development test was conducted by professionals using the intelligence development diagnostic scale for children (0–6 years old) in subjects in both groups. The children's intelligence development diagnostic scale, the Gesell Development Schedule, was revised by the Beijing Intelligence Development Group and used to assess the developmental quotients in each child's adaptive behavior, gross motor performance, fine motor movement, language development and individual social behaviors. A lower developmental quotient reflects a worse neurodevelopmental performance. A developmental quotient less than 85 (100–1SD) in more than two items was considered to be a poor neurodevelopmental outcome, whereas children with a developmental quotient of 85 or more in at least three subscales were considered to have a favourable neurodevelopmental outcome. Gesell Mental Developmental Scales were used to assess motor and cognitive performance at the corrected age of 12 months. These scales were performed by a neonatologist and a pediatric physiotherapist. These examiners were blinded to the infants' medical history.

### Statistical analyses

Numerical data are expressed as median with range (Kolmogorov-Smirnov test). Differences in numeric variables were assessed using the Mann-Whitney, Friedman's, or Kruskal-Wallis non-parametric two-tailed tests, with post-hoc analysis (Dunn's multiple comparison tests) where indicated. Fisher's exact test was used for the categorical variables. Multiple regression models were used to assess the influence of demographic and perinatal characteristics on CC16 and KL-6 levels. To validate the usefulness of KL-6 and CC16 in predicting neurodevelopmental outcome, receiver operating characteristic (ROC) curves were calculated and cut-off levels were determined when a significant result was obtained. Values of *p* < 0.05 were considered significant. All statistical analyses were performed using SAS 9.2 software (SAS Institute, Cary, NC, USA).

## Results

### Neonatal characteristics and neurodevelopmental outcome at 12 months of age

**Figure 1:**
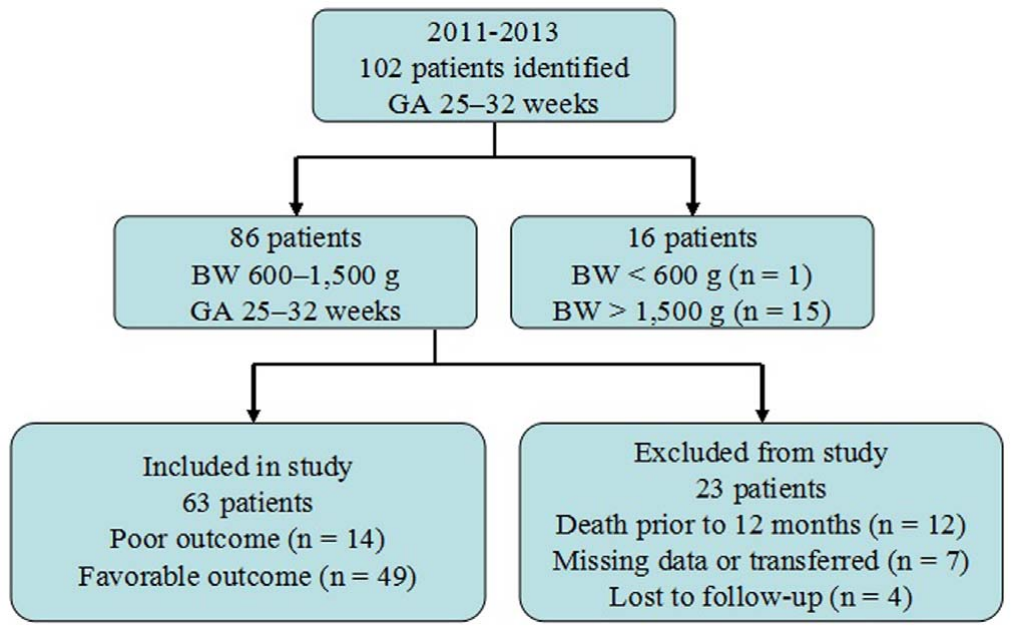
Flow chart showing enrollment of study subjects.

During the study period, 86 pre-term infants met the inclusion criteria. Among these infants, 12 died and 11 were lost to follow-up or transferred because the family moved away from the Hangzhou area or withdrew from the study. Finally, 63 infants were included in the study (Figure 1). 14 infants (gestational age of 28.5(28–30)weeks) had a poor neurodevelopmental outcome) and 49 infants (gestational age: 30 (29–31)weeks) had a favourable neurodevelopmental outcome (Table 1). Clinical characteristics of these two groups during the neonatal period are shown in Table 1I. No differences were found for Apgar score, cord blood pH, incidence of pre-natal steroid use, chorioamnionitis, surfactant use, sepsis, and necrotizing enterocolitis. Compared with infants with favourable neurodevelopmental outcome, those infants with poor neurodevelopmental outcome had significantly lower mean birth weight (*p* = 0.0005), gestational age (*p* = 0.0026), a higher incidence of mechanical ventilation ≥1 weeks (*p* = 0.04), BPD (*p* = 0.001), peri-ventricular–intraventricular haemorrhages larger than grade II (*p* = 0.03), PVL (*p* = 0.04), and steroid use for BPD (*p* = 0.0002). The head circumference and the Gesell score (in each item) in children with poor neurodevelopmental outcome was significantly lower than in those infants with favourable neurodevelopmental outcome.

### Levels of CC16 and KL-6 and neurodevelopmental outcome

At 7 days post-partum, the CC16 levels (274.4 ± 55.9 pg/ml) in infants with poor neurodevelopmental outcome were significantly lower than those levels in infants with favourable neurodevelopmental outcome (396.1 ± 103.3 pg/ml, *p*<0.0001, Table 2). Conversely, the KL-6 levels in infants with poor neurodevelopmental outcome (114.7 ± 17.3 ng/ml) were significantly higher than in those infants with favourable neurodevelopmental outcome (78.4 ± 20.0 ng/ml, *p*<0.0001). Multivariate logistic regression analysis with poor neurodevelopmental outcome as the dependent variable, revealed that higher KL-6 levels (adjusted odds ratio [aOR]: 1.12, 95% CI: 1.01–1.23; *p* = 0.020) and lower CC16 levels (aOR: 0.71, 95% CI: 0.51–0.98; *p* = 0.048) (Table 3) were independently correlated. In addition, use of steroids for BPD (aOR 41.73; 95% CI 1.47–632.98; *p* = 0.028) was independently associated with poor neurodevelopmental outcome after adjustment for confounding variables including gestational age, birth weight, incidence of BPD, mechanical ventilation ≥ 1 week, IVH, and PVL.

Plasma KL-6 levels were not significantly correlated with fine motor movements (r = −0.24, *p* = 0.05) and gross motor performance (r = −0.23, *p* = 0.06) but they showed a significant inverse correlation with head circumference (r = −0.31, *p* = 0.01), adaptive behavior (r = −0.35, *p* = 0.004) language development (r = −0.33, *p* = 0.008) and individual social behaviors (r = −0.35, *p* = 0.004) (Table 4). Plasma CC16 levels were not significantly associated with fine motor movements (r = 0.17, *p* = 0.16), gross motor performance (r = 0.25, *p* = 0.05) and head circumference (r = 0.23, *p* = 0.06), but showed a significant positive correlation with adaptive behavior (r = 0.33, *p* = 0.007), language development (r = 0.30, *p* = 0.01) and individual social behaviors (r = 0.34, *p* = 0.005).

**Figure 2:**
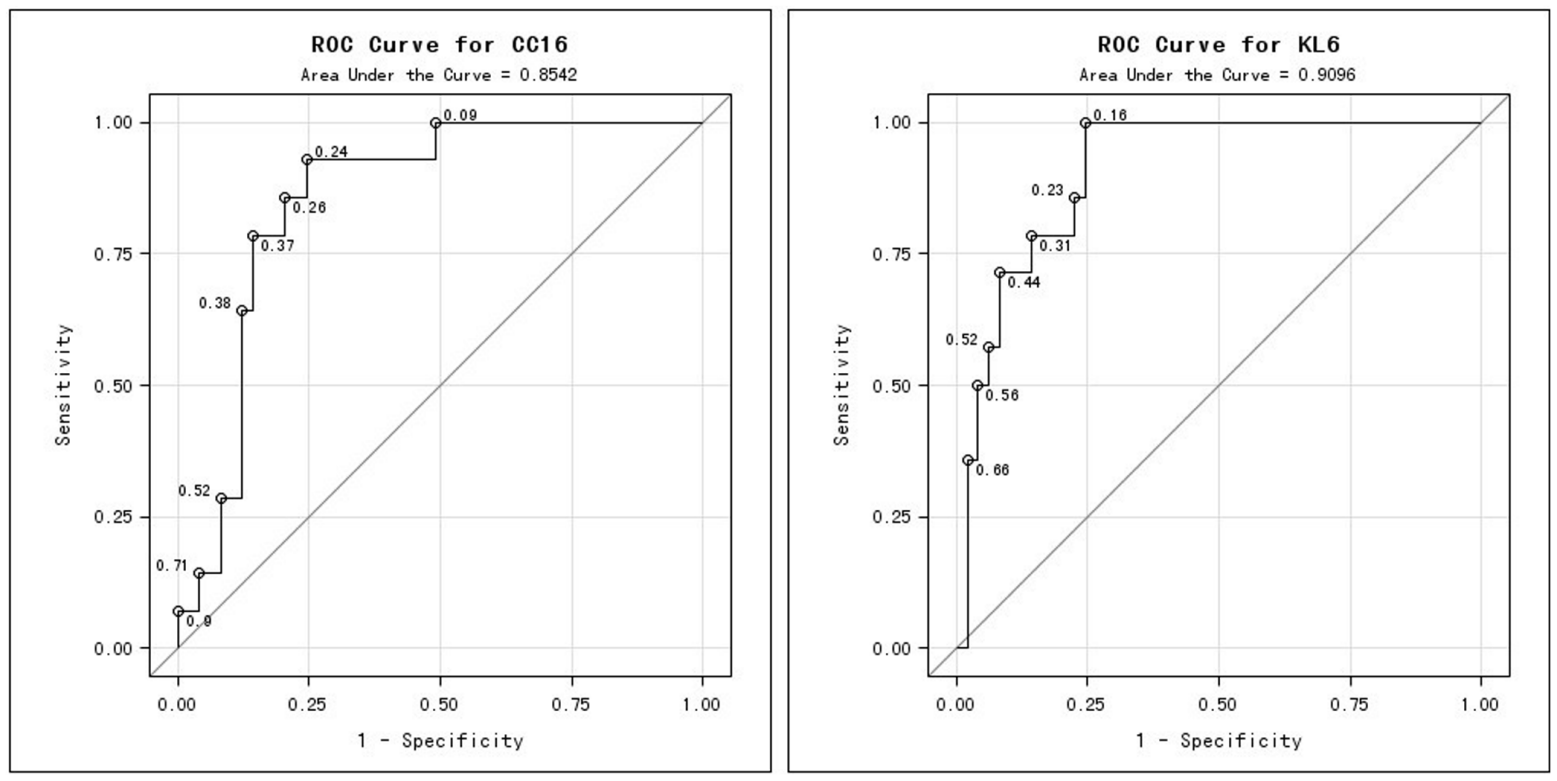
Receiver operating characteristic curves of plasma KL-6 and CC16 levels in predicting the occurrence of poor neurodevelopmental outcome.

The receiver operating characteristic curve for KL-6 and CC16 levels at 7 days post-partum had areas under the curve of 0.91 (95% CI 0.84–0.98) and 0.854 (95% CI 0.756–0.951), respectively (Figure 2). The computed cut-off values for KL-6 and CC16 relating to poor neurodevelopmental outcome were 90.0 ng/ml and 32.0 pg/ml respectively (Table 5). The sensitivity and specificity of KL-6 and CC16 for predicting poor neurodevelopmental outcome were 100% and 75.5%, and 92.8% and 85.7% (Table 5), respectively. Positive and negative predictive values of KL-6 and CC16 to predict poor neurodevelopmental outcome were 47.8% and 100%, and 65.0% and 97.6%, respectively (Table 5).

## Discussion

In this prospective cohort study, we found that there was a negative correlation between the KL-6 levels at 7 days in very pre-term infants and favourable neurodevelopmental outcome at 12 months of corrected age as determined by the Gesell scales after adjustment for confounding variables. In contrast, serum CC16 levels at day 7 seemed to be positively correlated with favourable neurodevelopmental outcome, although this was not statistically significant.

Elevated KL-6 and decreased CC16 levels respectively are valid and sensitive early indicators of respiratory diseases in infants, and predictors of BPD and its severity^5^,^6^,^7^,^8^,^11^,^12^,^13^,^14^. These parameters have also been proposed to mirror the intensity of perinatal inflammatory activity and oxygen toxicity^3^,^4^,^10^,^11^. Studies also linked both inflammation and infection in the pathogenesis of white matter injury (WMI) and cerebral palsy in pre-term infants^30^,^31^. An association between WMI and/or cerebral palsy with maternal, placental, or fetal infections^32^,^33^,^34^ with high levels of interleukin (IL)-1, IL-6 in amniotic fluid, and IL-6 in cord blood has been demonstrated^35^. Animal studies using inhalation of dust have shown a decrease in CC16 concentration, impaired anti-inflammatory potential, and resulted in early harmful effects not only to the respiratory tract but also to the nervous system^36^,^37^. Halatek *et al*^37^. found that the CC16 levels of bronchoalveolar lavage fluid in dust-exposed groups were significantly lower than in the control group. At all-time points (24 h, 7 days and 1 month), activity of pseudocholinesterase was found to be simultaneously increased. Increased pseudocholinesterase activity could suggest low-grade systemic inflammation due to induction of neurobehavioral effects, probably by changes in the “cholinergic anti-inflammatory pathway” in which acetylcholine modulates both immune response and neurotransmission^38^. Therefore, we speculate that exposure to pulmonary or systemic inflammatory disease resulted in lower CC16 and higher KL-6 protein levels which lead to persistent low-grade inflammation, which consequently resulted in induction of neurobehavioral effects. This may be due to changes in cholinergic anti-inflammatory pathways modulated by acetylcholine neurotransmission^37^. In addition, BPD is a recognized antecedent of cerebral palsy and adverse motor outcomes^39^,^40^,^41^,^42^. Skidmore *et al*.^39^ reported that cerebral palsy occurs more frequently (15% vs. 3.4%) in very low birth weight infants with BPD than those without BPD. Singer and colleagues demonstrated that BPD was a significant, independent predictor of poor motor outcome at 3 years of age after controlling for confounding variables and its association with a 10–12 point decrement in psychomotor developmental index scores^41^. Recently, Gagliardi *et al*.^42^ analysed the relationship between BPD and brain WMI in a large cohort of very pre-term infants admitted to 12 hospitals in northern Italy. They found an association between BPD and an increased risk of white matter damage^42^. Furthermore, Cornelie *et al*.^43^ found that poor neurodevelopmental outcome was associated with increased endogenous carbon monoxide in pre-term infants, which was identified as an early marker for predicting BPD in previous research^44^,^45^. Recurrent episodes of hypoxia in BPD patients may also affect neuronal organization, myelination and cellular apoptosis^46^. Additionally, the mechanism proposed for poor neurodevelopment of infants with BPD include postnatal corticosteroid use affecting brain growth. In our study, steroid use for BPD emerged as an independent risk factor for poor neurodevelopmental outcome. All these aspects taken together suggest that high levels of KL-6 and low levels of CC16 at early age are closely related to poor neurodevelopmental outcome and BPD in premature infants. Perinatal risk factors are considered to be key to the occurrence of bronchopulmonary dysplasia^47^ and poor neurodevelopmental outcome^48^. The first seven days of markers in serum samples reflect the various perinatal risk factors.

The limitations of the present study include the relatively small sample size, which make interpretation of the analyses at the population level very difficult since there could be small but clinically relevant differences in some of the outcome parameters. In addition, the Gesell score has certain subjectivity. Finally, our results are limited to neurodevelopmental assessment at 12 months corrected age, a relatively early developmental stage. Future research should assess larger cohorts and developmental age assessment extending to 18–24 months as this may corroborate our findings.

In conclusion, the present study has found that the majority of infants with poor neurodevelopmental outcome at 12 months of corrected age had increased both serum KL-6 and decreased CC16 levels at day 7 of life. KL-6 levels of more than 90.0 ng/ml and CC16 level less than 320.0 pg/ml at 7 days of life were highly predictive of a poor neurodevelopmental outcome at 12 months of corrected age.

## Author Contributions

X.M.H. conceived the idea and designed the research with Z.Q.Z. Z.Q.Z. analyzed the data and wrote the manuscript. Z.Q.Z., Y.X.Z., J.H.X. and H.L. supplied theoretical background for the explanation. All the authors contributed to discussion of the results.

## Figures and Tables

**Table 1:** Comparison of demographic and clinical characteristics between infants with and without poor neurodevelopmental outcome*

	Poor outcome (n = 14)	Favorable outcome (n = 49)	*p*-value
Birth weight, g	1140 ± 170.2	1310.8 ± 130.6	0.0005
Gestational age, weeks	28.5 (28–30)	30 (29–31)	0.0026
Male	7 (50.0%)	22 (44.9%)	0.74
Apgar score at 1 min	6.5 [5–8]	7 [5–7]	1.0
Apgar score at 5 min	7 [6–8]	7 [7–8]	0.81
Apgar score at 10 min	8 [7–8]	8 [7–8]	0.93
Vaginal delivery	4 (28.5%)	11 (22.4%)	0.64
Prenatal steroids	13 (92.8%)	45 (91.8%)	0.90
Maternal pregnancy-induced hypertension	4 (28.5%)	17 (34.7%)	0.67
Chorioamnionitis	4 (28.5%)	11 (22.4%)	0.64
Cord blood pH	7.30 ± 0.07	7.29 ± 0.08	0.75
Surfactant use	13 (92.8%)	34 (69.4%)	0.11
Mechanical ventilation ≥ 1 week	6 (42.8%)	8 (16.3%)	0.04
Sepsis	6 (42.8%)	15 (30.6%)	0.39
Necrotizing enterocolitis	6 (42.8%)	16 (32.6%)	0.48
Bronchopulmonary dysplasia	9 (64.2%)	6 (12.2%)	0.001
Intraventricular haemorrhages > gradeII	7 (50.0%)	10 (20.4%)	0.03
Periventricular leukomalacia	6 (42.8%)	8 (16.3%)	0.04
Steroids for BPD	8 (57.1%)	3 (6.1%)	0.0002

*Values are expressed as median [range] or number of individuals (percentage).

**Table 2:** Comparison of KL-6、 CC16 levels and scores of each item of Gesell Mental Developmental Scales between infants with and without poor neurodevelopmental outcome

	Poor outcome	Favourable outcome	*p*-value
KL-6 (ng/ml)	114.7 ± 17.3	78.4 ± 20.0	<0.0001
CC16 (pg/ml)	274.4 ± 55.9	396.1 ± 103.3	<0.0001
head circumference, cm	43.07 ± 0.9	43.68 ± 0.7	0.02
fine motor behavior	84.7 ± 8.8	90.6 ± 5.8	0.02
Large motor behavior	82.8 ± 8.1	95.1 ± 9.0	<0.0001
Adaptability behavior	79.0 ± 5.4	93.8 ± 10.5	<0.0001
Language behavior	78.0 ± 7.5	91.1 ± 9.4	<0.0001
personal social behavior	82.0 ± 6.7	93.0 ± 6.6	<0.0001

**Table 3:** Risk factors for poor outcome from multivariate logistic regression model

	aOR	95% CI	*p*-value
High KL-6^1^	1.12	1.01–1.23	0.020
Low CC16^1^	0.71	0.51–0.98	0.048

^1^Adjusted for birth weight, gestational age, mechanical ventilation ≥ 1 week, BPD, IVH, PVL and steroids for BPD.

**Table 4:** Associations of plasma KL-6 and CC16 levels with each item of Gesell Mental Developmental Scales and Head circumference

	Fine motor behavior		Gross motor behavior		Adaptability behavior		Language behavior		Personal-social behavior		Head circumference	
	r	*p*	r	*p*	r	*p*	r	*p*	r	*p*	r	*p*
KL-6	−0.24	0.05	−0.23	0.06	−0.35	0.004	−0.33	0.008	−0.35	0.004	−0.31	0.01
CC16	0.17	0.16	0.25	0.05	0.33	0.007	0.30	0.01	0.34	0.005	0.23	0.06

**Table 5:** KL-6 (ng/ml) and CC16 (pg/ml) cut-off levels for predicting poor neurodevelopmental outcome

				Predictive value (%)	
	Cut-off	Sensitivity (%)	Specificity (%)	PPV	NPV
KL-6 (ng/ml)	≥89.99	100	75.5	47.8	100
CC16 (pg/ml)	≤320.27	92.8	85.7	65.0	97.6
